# Influence of Cryopreservation on the Acrosome Reaction in Hucul Stallion Spermatozoa

**DOI:** 10.3390/ani15131915

**Published:** 2025-06-28

**Authors:** Monika Bugno-Poniewierska, Monika Bielecka, Natalia Pietras, Barbara Kij-Mitka, Zenon Podstawski, Bogusława Długosz

**Affiliations:** Department of Animal Reproduction, Anatomy and Genomics, University of Agriculture in Krakow, al. Mickiewicza 24/28, 30-059 Krakow, Poland; mbielecka918@gmail.com (M.B.); natalia.pietras@interia.pl (N.P.); barbara.kij-mitka@urk.edu.pl (B.K.-M.); zenon.podstawski@urk.edu.pl (Z.P.); boguslawa.dlugosz@urk.edu.pl (B.D.)

**Keywords:** stallion, sperm, acrosome reaction, cryopreservation, Hucul horses, CASA system

## Abstract

The Hucul horse is a Polish primitive breed with a small population size, which highlights the importance of preserving the genetic resources. The cryopreservation of semen is essential for creating gene banks, but its effect on the acrosome reaction in Hucul stallions has not yet been investigated. The acrosome reaction is one of the most important physiological events associated with the fertilization process. Therefore, our goal was to determine the level of acrosome reaction in chilled and frozen/thawed Hucul stallion semen using the FluoAcro test and the SCA^®^ semen analysis system. We found that semen cryopreservation significantly reduced sperm motility and was associated with an increased percentage of acrosome-reacted spermatozoa. It should be noted, however, that, in this case, there was no negative control, and the results may reflect acrosomal damage rather than the elicited responses. Further validation of the methods with equine sperm and the inclusion of a control are recommended.

## Simple Summary

The acrosome reaction is one of the most important physiological events associated with the fertilization process. The acrosome becomes more labile under reduced temperature conditions, and the contents of the acrosomal cap may be lost. The effect of cryopreservation on the acrosomal status in stallion spermatozoa, particularly for Hucul stallions, has not been studied. The aim of this study was to determine the level of acrosome reaction in chilled and frozen/thawed Hucul stallion semen. Our results showed a significantly higher proportion of acrosome-reacted spermatozoa in frozen/thawed semen compared to chilled semen. In chilled semen, most of the acrosomes remained intact, suggesting the preserved potential for fertilization. In cryopreserved semen, a greater number of spermatozoa had reacted acrosomes, which in turn causes a significant reduction in sperm functionality. These findings suggest that cryopreservation may negatively affect acrosomal integrity, and further studies using appropriate controls are necessary.

## 1. Introduction

Hucul horses are classified as Polish primitive breeds, and their numbers are very limited, so it is important to protect them and to take actions to secure the genetic resources from this breed. However, due to the fact that the use of assisted reproductive technologies (ARTs) in the breeding of these animals is allowed only in exceptional cases, and only after obtaining the consent of the Stud Book Commission, semen from Hucul stallions remains an innovative research topic. Therefore, there is very little research on the semen of stallions from this breed. In the sperm bank of the University of Agriculture in Krakow, we have created a collection of frozen semen for this breed, and preliminary research has shown that the sperm of some Hucul stallions are sensitive to the cryopreservation process [1].

Sperm cryopreservation is still one of the most important ARTs. It allows to use insemination doses without any space or time limit. In addition, it is important in the context of securing genetic material from valuable individuals in a small population [1]. The use of cryopreserved semen may result in a reduced pregnancy rate, which is directly related to reduced sperm quality parameters [2]. The cryopreservation of semen may impair the process of capacitation and, consequently, the acrosome reaction, which is a process necessary for the proper fertilization process. The acrosome reaction in equine spermatozoa is known to be more difficult to induce compared to other species, and substances such as calcium ionophores, progesterone, or zona pellucida glycoproteins have been used in various protocols to trigger this reaction [3].

Due to the fact that the cryopreservation process can have a negative impact on the quality of sperm, a detailed analysis is necessary. Computer-Assisted Semen Analysis (CASA) allows for a quick and objective assessment of a large sperm count in a short period of time, and is a source of very detailed data characterizing the dynamics of male gametes, their viability, and morphology. An undeniable advantage of such tests is the fact that the standardization of equipment settings that prevents erroneous results allows for a reliable comparison of the semen evaluation results obtained in different laboratories [4]. CASA quality provides a richer and more detailed characterization of sperm motility compared to the conventional microscopic method. A more accurate description of the motor properties opens up opportunities for a more accurate estimation of the fertilizing capacity of spermatozoa [5,6]. Manufacturers of the SCA^®^ system also offer the opportunity to evaluate the success of the acrosome reaction. This reaction must be initiated by the process of capacitation during which sperm acquire the ability to fertilize. In vivo capacitation takes place in the female reproductive tract [7]. An important feature of capacitation is that it is species specific. Sperm introduced into the reproductive tract of a female of another species are unable to undergo capacitation. Components produced by the epithelial cells of the uterus and fallopian tubes cause changes in the sperm cell membrane. Capacitation-inducing factors, such as steroid sulfatase, serum-derived sterol acceptors, and phospholipase or glycosaminoglycans (GAG) secreted in the uterus and fallopian tubes, are able to remove substances covering the sperm membrane [8,9,10]. The release of acrosomal enzymes allows the sperm to penetrate into the egg cell [11,12].

Despite over 70 years of research on acrosome reaction, the search for the molecular basis of this process is still of interest to many scientists [12]. The first independent study by two teams in 1951 confirmed that rabbit and rat sperm need to spend about 2 h in the female reproductive tract to acquire the capacity to fertilize an oocyte [13,14]. This provided the basis for many years of research, and confirmed that sperm need time to undergo the process of “capacitation”, in order to achieve fertilization potential.

Capacitation is a prerequisite for the irreversible acrosome reaction. When the sperm head fuses with the zona pellucida, an acrosome reaction is initiated in response to the glycoproteins of the zona pellucida (ZP1, ZP2, ZP3) and to the chemical signal from progesterone. Progesterone causes an influx of calcium ions into the cells, which is necessary to trigger the reaction. The sugar residues of the ZP3 and ZP2 proteins participate in sperm binding on the surface of the zona pellucida. The ZP3 glycoprotein is recognized by β-1-4-galactosyltransferase (GalTase) and the p95 protein, the SP56 protein, and spermadhesins, i.e., sperm membrane proteins. As a consequence of the connection of ZP3 with the sperm, the spatial arrangement of the receptor proteins changes. Intracellular pathways are activated, leading to the opening of calcium channels and the influx of Ca^2+^ ions into the cell. In the next step, the acrosome reaction occurs, activating enzymes that facilitate penetration of the zona pellucida. These enzymes are proteinase, esterase, phospholipase, acid glycohydrolase, phosphatases, and collagenase. The inner acrosome membrane is exposed, and hyaluronidase and acrosin bind to it [15,16]. Acrosin has the ability to modify other acrosomal proteins, and participates in the binding of sperm receptors to the ZP2 glycoprotein [17,18]. The acrosome reaction is an exocytotic process and an absolute condition for successful sperm-egg interaction.

Studies conducted on human semen show that, during cryopreservation, and thus under conditions of decreasing temperature, the acrosome becomes more sensitive. As a consequence, the contents of the acrosomal cap are lost. Sperm with a disturbed acrosome reaction cannot fuse properly with the zona pellucida, so they are unable to fertilize [19,20,21,22]. In horse breeding, the cryopreservation process itself is well known, but due to the high inter-individual variability, as well as to the particular sensitivity of some stallions’ semen to the freezing and thawing process, the research to optimize this process is still ongoing [18]. As the effect of cryopreservation on the acrosomal status in Hucul stallion spermatozoa has not been studied, the aim of our work was to determine the level of acrosome reaction in chilled and frozen/thawed semen of Hucul stallions using the FluoAcro test and the SCA^®^ automatic semen analysis system. The research hypothesis assumed that the cryopreservation process would increase the number of spermatozoa in which the acrosome reaction occurred prematurely.

## 2. Materials and Methods

### 2.1. Material

The nine stallions from which the semen (no. of individuals I–IX; Table 1) was obtained came from the Hucul Horse Stud in Gładyszów, where standard breeding methods are used. The horses were maintained under uniform environmental and fed ad libitum with grass and/or hay and had ad libitum access to water. All horses were kept in a free-range system with full welfare conditions to limit the impact of these factors on the semen parameters. In addition, qualified personnel were responsible for collecting the semen. The animal study protocol was approved by the Institutional Commission for Animal’s Welfare of the Department of Animal Husbandry and Biology. All animal procedures were approved by the Local Ethics Committee in Krakow, Poland, resolution No. 98/2021, in accordance with the legal regulations of the European Union.

Semen was collected at the horses’ residence (Hucul Horse Stud in Gładyszów), and ejaculates were initially evaluated and secured for transport in a mobile andrology laboratory (AndroBus), maintaining stable temperature, laboratory conditions, and appropriate equipment. A single ejaculate was collected from each of nine stallions (aged from 5 to 11 years old) using an open-ended artificial vagina (Ar Model—University of Agriculture in Krakow, Poland) which allowed the separation of the sperm-rich fraction from the gel fraction and/or to avoid contamination of the semen with blood, urine, or bacteria. All of the semen collections were made in February. The collected ejaculates were diluted with the INRA 96 diluent (IVM Technologies, Marseille, France) at a temperature of 37 °C, in a 1:1 ratio and secured in sterile 50 mL falcons. After that, semen samples were transported at +4 °C to the laboratory, where they were subjected to a detailed evaluation and cryopreservation. The time between semen collection and the start of the experiment was 24 h, and it was necessary to transport the semen from the stud to the laboratory and to start the procedures in controlled time and laboratory conditions.

Sperm analyses were performed using the SCA^®^ 6.6.15 system (Microptic S.L., Barcelona, Spain), which is an advanced computerized semen analysis (CASA) system that includes modules for the assessment of motility, morphology, concentration, and acrosome reaction.

### 2.2. Evaluation of Sperm Concentration and Motility

Sperm concentration and motility were determined three times, immediately after semen collection and again when starting the experiment in the laboratory (24 h after collection), before and after semen cryopreservation, and immediately before the assessment of the acrosome reaction. Heated to 37 °C, aliquots of semen (2 µL) were placed on a GoldCyto^®^ slide (TK Biotech, Warsaw, Poland) and analyzed under a light microscope (equipped with a heated table) at approximately 10× magnification with phase contrast optics (Nikon Eclipse E200, Nikon, Tokyo, Japan). The “motility” module of the SCA^®^ system was used for the analysis determining the total and progressive movement in each semen sample. Depending on the concentration, from 3 to 10 research areas were recorded, observing a minimum of 500 spermatozoa.

### 2.3. Semen Cryopreservation

After the assessment of sperm concentration and motility, the semen was cryopreserved. Diluted ejaculates were centrifuged for 7 min at 2500 rpm. Following centrifugation, the supernatant from each tube was removed and discarded. The sperm pellets were suspended to 120 million sperm/mL with the Spectrum Red extender (SBS CryoSystem Spectrum Freezing Extender; Insatex-MT, Poznan, Poland) in an amount appropriate for the concentration and mixed. Semen prepared in this way was transferred to 0.25 mL straws using a semi-automated method for filling straws (SFS 133, cat. no. 13133/0135 Minitube, Tiefenbach, Germany). The straws were equilibrated for 30 min in a refrigerator (4 °C). After that, the straws were placed on a Styrofoam float and cooled in liquid nitrogen vapor for 15 min before being transferred directly to liquid nitrogen and stored until analysis.

### 2.4. FluoAcro^®^—Sperm Functional Test

The FluoAcro^®^ kit (Microptic S.L. Barcelona, Spain) was used to diagnose the acrosome reaction in the stallion sperm, according to the manufacturer’s instructions.

Cryopreserved semen was thawed at 37 °C for 30 s. For each individual, 2 straws were used, with their contents combined after thawing by transfer to a 1.5 mL Eppendorf tube. Similarly, 0.5 mL of cooled semen was transferred into a 1.5 mL Eppendorf tube. Two test samples (chilled semen and frozen/thawed semen) were obtained for each individual. In the next step, 1 mL of PBS (Sigma-Aldrich; Merck Life Science, Poznan, Poland) was added to each tube followed by centrifugation for 8 min at 2000 rpm. After the supernatant was aspirated, 1 mL GM501 SpermAir (Gynemed GmbH & Co. KG, Sierksdorf, Germany) at 37 °C was added to 300 µL washed semen and incubated for 25 min at 37 °C. Then 10 µL of Solution 1 (a Ca-based medium to support the acrosome reaction) was added, and the sperm suspension was incubated at 37 °C for a further 15 min. After incubation, the reaction was stopped by adding 100 µL 70% ethyl alcohol (EtOH). A small volume of each sperm suspension (10 µL) was then spotted in three places on the FluoAcro slides using the template included in the FluoAcro kit, and the preparations were dried for 10 min at 37 °C. The slides were then placed in 95% EtOH at 4 °C for 30 min in the dark and then air dried. The Acrosome Staining procedure was started when the preparations had dried. An amount of 20 µL of Solution 2 (PNA staining solution) was applied to each semen spot on the slide and incubated for 5 min at room temperature before the addition of a further 30 µL of Solution 2 and incubation for 75 min in a moist chamber in the dark, after which the slides were rinsed twice in PBS in the dark and dried. For each dried drop, 10 µL of final working Solution 3 (Basic Hoechst type working solution) was added and incubated for 10 min in the dark. In the next step, the slides were dipped very slowly in distilled water for 1 s and dried in the dark at room temperature. Prior to fluorescence microscopy, 15 µL of Solution 4 (Anti-fading fluorescence medium) was applied (in the dark) to the slides and covered with a coverslip. Acrosome reaction analysis was performed using a Nikon Eclipse E200 fluorescence (Nikon, Tokyo, Japan) microscope with an integrated SCA^®^ semen quality assessment system. The evaluation was carried out by the SCA^®^ Acrosome reaction module according to the manufacturer’s instructions using fluorescence light specific for DAPI and FITC dyes. Intact acrosomes were shown as bright green/blue and only blue if there was no acrosome present. In that case the spermatozoa were counted as “reacted”. The most typical patterns are the full acrosome (bright green) or a green band at equatorial segment (reacted) and blue (reacted). For each analysis of the acrosome reaction, a minimum of 600 spermatozoa were examined per stallion (200 per field, 3 replicates).

### 2.5. Statistical Analysis

The statistical significance of the results was determined using the SAS Enterprise Guide 8.1 software. Normality of distributions were examined by Kolmogorov–Smirnov test. The level of significance of the Student’s *t*-test was determined for *p*-value = 0.05. The results for the acrosome reaction and motility were analyzed using the sign test. A linear regression equation was used to determine the relationship between motility and the level of acrosome reaction, and the correlation was determined using the Spearman correlation coefficient in the OriginLab 2021b program.

## 3. Results

### 3.1. Sperm Motility

For each stallion, sperm motility (total and progressive) was assessed both in chilled semen (before cryopreservation) and after cryopreservation and thawing of the straws, just before starting the procedure to determine the level of acrosome reaction. The results are presented in Table 1. Semen cryopreservation resulted in a significant reduction in sperm motility (Pr. ≥ |S| = 0.0156). Significant inter-individual differences were also noted—stallion VI had the best post-thaw sperm motility, while stallions II and IV had the worst post-thaw sperm motility.

### 3.2. Acrosome Reaction

The FluoAcro test, which is a functional test for sperm analysis, was used to study the acrosome reaction. This test distinguishes between spermatozoa with an intact acrosome and those with a reacted acrosome, based on fluorescent staining. The classification of sperm as having undergone acrosome reaction was based solely on the staining pattern following chemical induction. As a negative control (uninduced sample) was not included, this limited the ability to specifically distinguish induced acrosomal reactions from acrosomal damage caused by cryopreservation.

**Figure 1:**
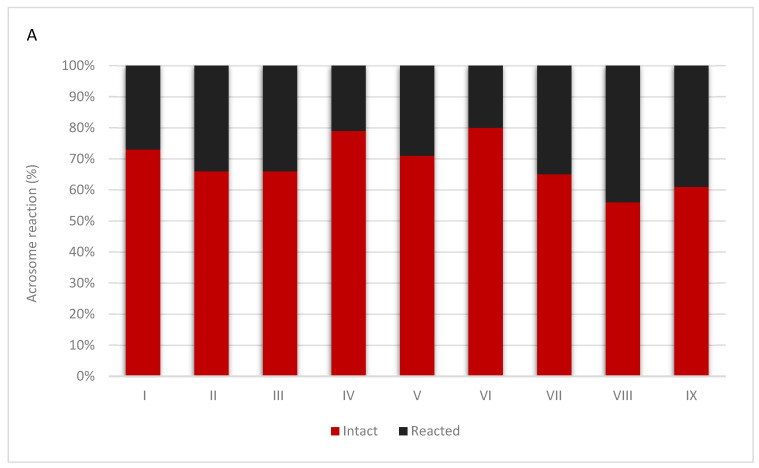

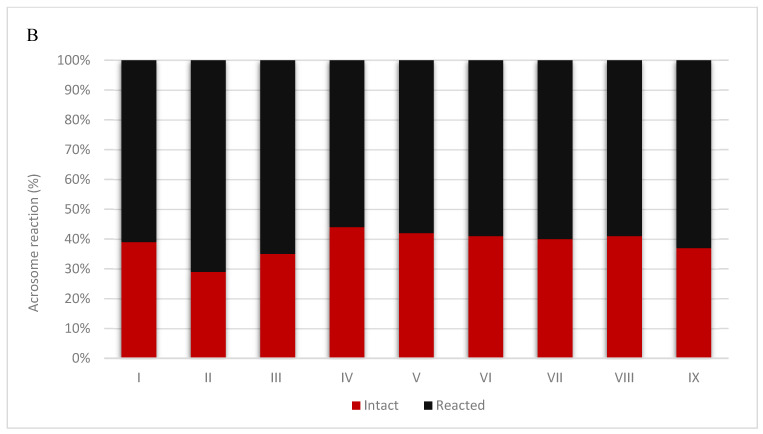
Results of the acrosome reaction in refrigerated semen (**A**) and frozen/thawed semen (**B**) collected from nine individuals. The value of intact and reacted spermatozoa of each individuals are shown as a percentage.

**Figure 2:**
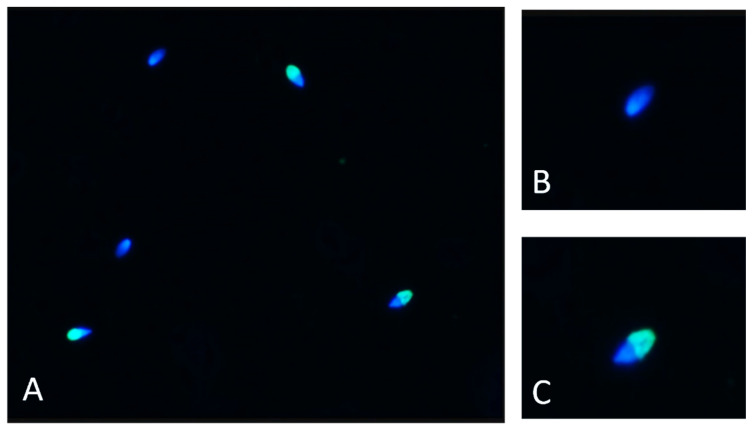
(**A**) Sample picture of Hucul horse spermatozoa after undergoing the acrosome reaction; green fluorescence shows acrosome intact; blue color indicates acrosome reacted. (**B**) Sample picture of Hucul horse acrosome-reacted spermatozoon. (**C**) Sample picture of Hucul horse intact spermatozoon. The images were taken using 100× magnification. DAPI and FITC filters were used.

The study of the acrosome reaction in the sperm of Hucul stallions made it possible to compare the results of this reaction between chilled semen and semen after thawing. The obtained results are presented in Figure 1. Figure 2 present spermatozoa after undergoing the acrosome reaction.

**Figure 3:**
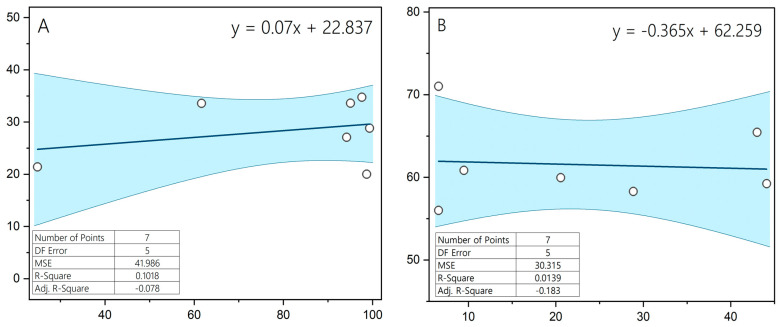
Relationship between the level of acrosome reaction and sperm motility in chilled semen (**A**) and thawed Hucul horses semen (**B**).

There was no statistically significant relationship between the incidence of acrosome reaction and sperm motility, in either chilled semen or after cryopreservation (both *p* > 0.05), and the correlation between these parameters was low (r = 0.04; r = 0.07) (Figure 3).

## 4. Discussion

Hucul horses are considered to be a Polish conservation breed. The genetic material derived from its representatives is valuable due to the limited number of this breed. The use of artificial insemination is allowed only in special cases, and only after obtaining the consent of the Stud Book Commission. Due to this, the usefulness of semen from Hucul stallions is poorly understood, and research work conducted on their semen is an extremely interesting scientific issue.

To maximize the likelihood of successful sperm entry into the oocyte and pronuclear fusion, a significant number of sperm must reach the zona pellucida so that only one can penetrate it [12]. After a spermatozoon attaches to the zona pellucida, it must undergo the acrosome reaction to be capable of reaching and then entering the oocyte. The zona pellucida is digested by the compounds contained in the acrosome, which facilitates the spermatozoon’s passage. On the other hand, if sperm cells have already lost their acrosomes prior to insemination, as a result of cryopreservation, this may reduce the fertilization potential of the specimen [19,20,21,22].

The cryopreservation process may negatively affect sperm quality parameters and be associated with reduced fertilization potential. Increasingly, CASA systems enable fast and precise analysis, including parameters such as motility, concentration, viability, and even acrosomal reaction. The use of this tool in the assessment allows us to maintain maximum reliability of the performed tests [4].

Researchers studying changes in semen after cryopreservation have shown that this process impairs sperm motility and vitality as well as the ability to penetrate the zona pellucida. The reason for the reduced fertilizing capacity of these spermatozoa is mainly due to a reduction in the proportion with normal, intact acrosomes [23,24]. Studies of human semen have shown that, during cryopreservation, and thus under the conditions of decreasing temperature, the acrosome becomes more sensitive and its contents are lost. Spermatozoa from men with a disturbed acrosome reaction cannot fuse properly with the zona pellucida and are unable to fertilize on their own. However, they can be used for intracytoplasmic sperm injection (ICSI).

Although the impact of the cryopreservation process on the basic parameters of stallion semen is relatively well known, high inter-individual and inter-breed variability is still an interesting subject of research [1,25,26,27,28,29]. Furthermore, studies on the functionality of acrosomes in frozen/thawed stallion spermatozoa have not been conducted to date.

Cryopreservation of stallion semen, as in the case of human semen, causes a wide spectrum of changes within the sperm head, including affecting its acrosome [12]. In addition, sperm motility significantly decreases after the ejaculate has been cryopreserved [30,31,32,33]. Studies on the motility of cryopreserved horse semen have shown that the total and progressive sperm motility is higher in chilled semen at both 24 and 48 h post-ejaculation compared to all cryopreserved samples using the INRA 96 extender and cryoprotector and the CryoMax LE (IMV Technologies, Saint-Ouen-sur-Iton, France) [2,34]. The results obtained in the present study concerning sperm motility showed a significant decrease in total sperm motility in cryopreserved semen in relation to chilled semen, with only two out of nine stallions having a total motility above 40%. This means that the sperm motility decreased after cryopreservation, which is consistent with most of the current literature. Earlier research conducted by Bugno-Poniewierska et al., concerning the basic semen parameters (i.e., sperm motility, vitality, and morphology) of 12 Hucul stallions, showed similar results before and after cryopreservation [1]. There was no significant difference in the percentage of motile sperm in the diluted and frozen semen groups (47% vs. 42%, respectively, *p* = 0.3372). However, there was a difference in the proportion of progressively motile spermatozoa, which was significantly higher for thawed semen samples (15% versus 7% for diluted fresh semen). It should be noted, however, that the cryopreservation process in that study was carried out immediately after semen collection; whereas, in the current study, cryopreservation was performed on semen that had been chilled for 24 h. This procedure allowed for the comparison of the effect of the cryopreservation process itself on the level of acrosome reaction in both tested samples. The 24-h interval between the collection of ejaculates and the performance of further methodological stages enabling the examination of acrosome reaction was dictated by the necessity of transporting ejaculates from the stud (where the semen was collected) to the laboratory, where it was possible to conduct the methodology in appropriate conditions using specialist equipment.

The current study is the first to investigate the effect of semen cryopreservation on the occurrence of the acrosome reaction in Hucul stallions, and gave similar results to those observed in humans. We showed a significant decrease in sperm motility, which ranged from 24.86% to 99.36% before freezing, and from 6.59% to 44.10% after freezing. The proportion of spermatozoa with an intact acrosome before cryopreservation ranged from 55.50% (stallion VIII) to 80% (stallion VI). This was decreased significantly after freezing and thawing, and ranged from 29.08 (stall II) to 40.76 (stallion VI). We also observed inter-individual variability between individual stallions, in terms of both sperm motility and the proportion of spermatozoa with an intact acrosome.

We found a low correlation between motility and acrosome reaction in both chilled semen and after cryopreservation, (r = 0.04 and r = 0.07, respectively). This result differs from the other studies, which found a correlation between sperm motility and vitality and the proportion of spermatozoa with intact acrosomes [35,36]. It should be noted, however, that the semen analyzed in these studies was from men, while our study is the first to determine the extent of intact acrosomes in stallion spermatozoa. The absence of correlation between motility and acrosome reaction observed in this study contrasts with the findings in human studies, where intact acrosomes are more frequent in motile sperm. However, our analysis focused on induced acrosome reaction, not baseline acrosome integrity, and the lack of a negative control prevents conclusive interpretation. Future studies should include assessments of acrosome integrity alongside reaction inducibility.

Our results clearly showed the negative impact of cryopreservation on acrosomal quality. In chilled semen, most of the acrosomes remained intact, which means that they have the ability to fertilize the oocyte. In cryopreserved semen, spermatozoa had largely lost their acrosomes, which in turn causes a significant reduction in sperm functionality. These data suggest the need to develop new sperm cryopreservation strategies that may improve the effectiveness of this technique in stallions by reducing or preventing acrosome loss.

The results presented by other authors show that the use of cryopreserved stallion semen has increased in recent decades and is closely related to the routine use of artificial insemination (AI) in equine reproduction [37,38]. Although artificial insemination is commonly used in most horse breeds, natural mating is used in the Hucul horse breed. Therefore, the current state of knowledge about the functionality of Hucul stallion semen and its quality after the cryopreservation process is very limited. However, cryopreservation allows for the preservation of genetic material and enables its use in the future, as well as increasing the biodiversity of small populations, which is of great importance in terms of creating banks of biological/genetic material.

## 5. Conclusions

Semen cryopreservation significantly reduced sperm motility and was associated with a higher proportion of acrosome-reacted sperm, which may reflect structural damage or non-specific activation. Further research into the effect of cryopreservation on the membrane and acrosome is necessary. It should be noted that there was no negative control group in this study. Therefore, these results may be the result of the methods involved in handling and processing, and may not be unique to the Hucul stallion. Further studies that include adequate controls are needed to validate these findings relative to the Hucul stallion.

## Figures and Tables

**Table 1:** Results of sperm motility (motile/progressive) analysis in chilled and thawed semen.

No. of Individuals	Age in Years	Motility	
		Chilled Semen Motile (%)/Progressive (%)	Thawed Semen Motile (%)/Progressive (%)
I	8	94.20/37.57	9.49/1.79
II	8	95.05/31.55	6.59/0.93
III	22	61.67/12.18	43.00/10.36
IV	8	24.86/4.67	6.59/0.93
V	11	99.36/54.7	28.86/4.67
VI	17	98.71/52.36	44.10/11.98
VII	11	97.63/42.62	20.56/4.15
VIII	12	90.82/38.97	33.91/6.56
IX	14	93.07/40.03	29.59/5.11

## Data Availability

All data presented in this paper are available from the corresponding author.
